# Negative symptoms assessment in early intervention settings: Implications for early identification and treatment

**DOI:** 10.1192/j.eurpsy.2021.124

**Published:** 2021-08-13

**Authors:** L.B. Glenthøj, M. Nielsen, M. Nordentoft

**Affiliations:** 1 Copenhagen Research Centre On Mental Health, Mental Health Centre Copenhagen, University of Copenhagen, copenhagen, Denmark; 2 Centre For Clinical Intervention And Neuropsychiatric Schizophrenia Research, Cins, Glostrup, Denmark, Mental Health Centre Glostrup, Glostrup, Denmark

**Keywords:** Assessment negative symptoms, Negative symptoms early psychosis, Functional outcome early psychosis

## Abstract

Negative symptoms are a core feature of schizophrenia spectrum disorders associated with poor outcomes such as low remission rates and impairments in daily functioning and quality of life in early psychosis. The assessment of negative symptoms in early psychotic disorders is predominantly conducted by use of first-generation scales such as the PANSS and the SANS, along with the SIPS and CAARMS for the psychosis clinical high-risk (CHR) state. Following the progressed conceptualization of negative symptoms, it has, however, been recognized that these scales suffer important methodological limitations. This warrants a use of second-generation scales such as the Brief Negative Symptom Scale (BNSS) and the Clinical Assessment Interview for Negative Symptoms (CAINS) in early intervention settings in order to achieve a more accurate assessment of the negative symptom complex. Advancing the assessment of negative symptoms in early psychosis may also guide more targeted intervention approaches aimed at improving functional outcome. Albeit recognizing that negative symptoms constitute an important barrier to a good functional outcome in psychotic disorders, few studies have directly aimed at alleviating negative symptoms in early psychosis. Meta-analytical evidence does, however, exist on the efficacy of the combined treatment modalities incorporated in Early Intervention Services (e.g. intensive and assertive case management, family involvement etc.) in reducing negative symptoms in first-episode psychosis. Evidence on the effect of interventions for improving negative symptoms in the CHR state is lacking. Developing targeted, and possibly more individualized negative symptoms treatment approaches, constitute an essential future research area.

**Disclosure:**

No significant relationships.

